# Integrating laser assisted Micro-EDM for development of micro-features: A hybrid approach

**DOI:** 10.1016/j.isci.2026.116653

**Published:** 2026-07-07

**Authors:** Vikas Kanake, B. Rajiv, Kedarnath Chaudhary

**Affiliations:** 1 COEP Technological University, Pune 411005, India

**Keywords:** Micro-Electric Discharge Machining, Micro-EDM, Hybrid, Laser Machining and biomaterial, Laser assisted micro EDM, LAMEDM

## Abstract

Non-traditional machining employs both laser and micro electrical discharge machining (EDM) processes to produce intricate shapes and handle difficult-to-cut hard materials without direct contact. Both technologies are prominent for drilling operations and are used in many sectors, such as aviation, automobiles, and medical. While considering laser and micro EDM machining processes have their own sets of limitations and advantages. Micro-EDM provides high surface quality, whereas laser is a significantly faster machining process. However, there is a limitation of traditional methods in fabricating micro features such as multiple electrodes, needles, and holes. Laser-assisted micro EDM (LAMEDM) facilitated the fabrication of such features precisely. This study also focuses on the biocompatible material, such as Titanium alloy and magnesium alloy, which are involved in the fabrication of micro-features varies around 70 μm–90 μm, and also achieved a high aspect ratio up to 7. Moreover, this study also addresses problems related to microelectrode breakage during insertion and removal, which can result in toxicity and skin irritation.

## Highlights

• Hybrid laser-micro-EDM fabricates multi-micro-electrode arrays
• It overcomes micro-EDM limits for high-density complex micro-features
• Biocompatible Ti and biodegradable AZ31 Mg alloys are micromachined
• AR 6–7 micro-structures achieve controlled spacing and improved morphology

## Graphical abstract

**Figure:**
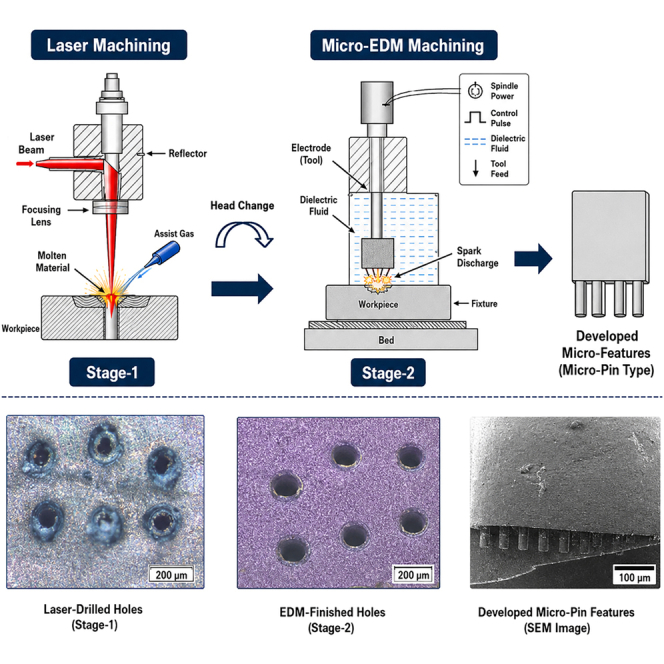

## Teaser

Laser; Physical pattern formation; Applied sciences

## Introduction

**Figure 1:**
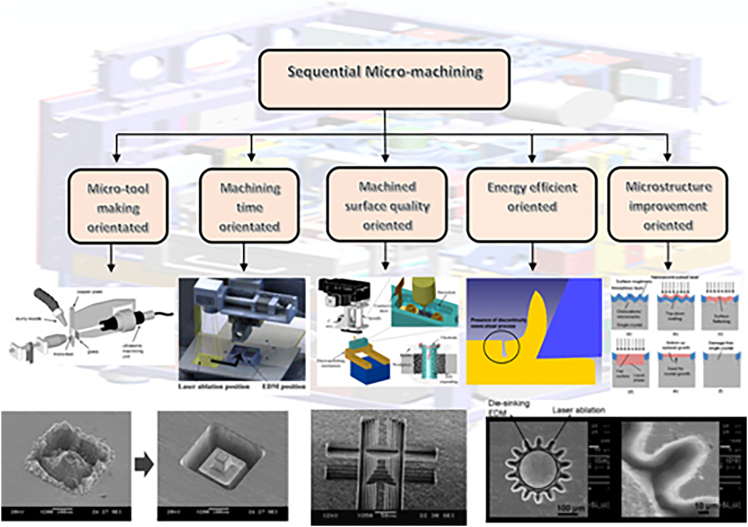
Applications of micro machining Representative applications of micromachining in sequential micro-machining processes. The figure illustrates micro-tool-making-oriented, machining-time-oriented, machined-surface-quality-oriented, energy-efficient-oriented, and microstructure-improvement-oriented applications relevant to precision manufacturing.

Micromachining with laser and electric discharge technique is an advanced trend in the world of precision manufacturing in industrial field such as the aviation sector, automobile sector, and medical sector, as shown in Figure 1.^1^ Both techniques target the difficult-to-cut hard material; specifically, Laser cutting gives the flexibility of rapid fabrication, and electrical discharge machining (EDM) technique gives the critical machining with precision. Nowadays, stainless steel, cobalt chromium, titanium alloy, nitinol, and magnesium are widely used for biomedical applications.^2^ Out of these materials, titanium alloys show excellent anti-corrosion and better acceptance property.^3^ Other than titanium, magnesium alloy is a bioactive material that has the property of biodegradation, which is dissolvable in the human body. Ti-6Al-4V super alloy is difficult to machine since it has poor machinability in the majority of conventional machining operations.^4^^,^^5^^,^^6^ Using non-conventional machining techniques such as micro-electro discharge machining and laser, which gives the optimal machining output.

**Figure 2:**
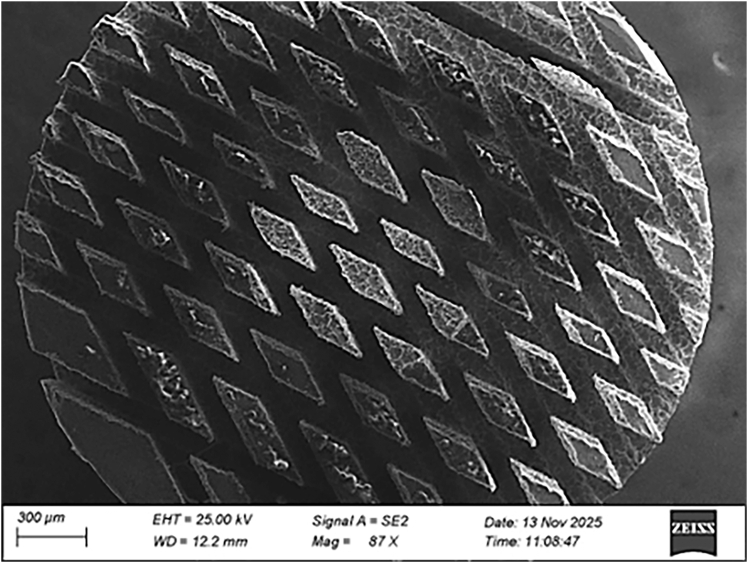
Multi-micro electrode fabricated using Wire EDM Scanning electron microscopy image shows a multi-micro-electrode array fabricated using wire electrical discharge machining. The figure represents a conventional approach for producing micro-electrode arrays and highlights the relevance of micro-electrode fabrication for biomedical and sensing applications.

Laser-assisted micro-EDM (LAMEDM) is a manufacturing process that combines the precision of laser ablation with the material removal capabilities of micro-EDM (micro-EDM) to produce micro-scale features on a range of materials. One of the studies related to micro electrode array fabrication with the wire EDM technique used for recording the brain activity of a mouse resulted in capturing the neural signal.^7^ Most of the cases, wire EDM materials are used as tungsten, brass, and copper. The material of these wire debris sticks on the surface of the electrode, which can create adulteration, so such an implant is not suitable for biological activity. The resulting micro-electrode can be used for a range of applications, including bio-sensing, drug delivery, and neural stimulation. The patch can be designed with different shapes and sizes of electrodes, allowing for targeted stimulation or sensing of specific areas^8^ as shown in Figure 2.

Micro-needles can be made from a range of materials, including silicon, glass, metal, and polymers. They are often designed to be minimally invasive and painless, making them useful for applications where traditional needles may be too uncomfortable or impractical.^9^

**Figure 3:**
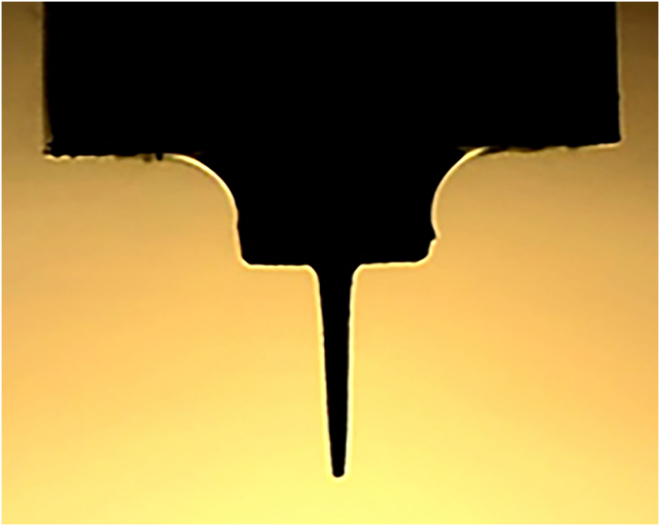
Micro-needle fabricated using laser-assisted Micro EDM Representative image of a micro-needle fabricated using laser-assisted micro-electrical discharge machining. The figure illustrates the possibility of producing high-aspect-ratio micro-features suitable for biomedical applications such as transdermal delivery, sensing, and minimally invasive devices.

One application of micro-needles is in transdermal drug delivery, where they are used to penetrate the skin and deliver medication directly to the bloodstream, as shown in Figure 3.

Micro-needles can be used to deliver a wide range of drugs, including insulin for diabetes, vaccines, and other medications.^10^ In addition to drug delivery, micro-needles are also used in research settings to sample fluids from the body. For example, micro-needles can be used to collect blood samples, measure glucose levels in interstitial fluid, or collect cerebrospinal fluid from the brain.^11^

Micro-needles can also be used for aesthetic purposes, such as skin rejuvenation, where they are used to create tiny channels in the skin that stimulate the production of collagen and improve the appearance of fine lines and wrinkles.^12^ While micro-needles have many advantages, such as their minimally invasive nature and their ability to deliver drugs through the skin, there are also some potential drawbacks to their use. Micro-needles can be fragile and may break during use, which can be a safety concern for both the patient and the user. Micro-needles can cause skin irritation or inflammation, especially if they are used repeatedly in the same area.^13^ During the EDM process, it is found that the use of electrode material (copper, brass, and tungsten) affects the biocompatibility of medical implants by the deposition of a small amount of tool material, which creates an inflammatory response and toxicity.^14^^,^^15^ The utilization of multi micro electrodes made from biodegradable magnesium alloy can mitigate the issue of potential breakage during use. If the electrode happens to break during insertion, there is no risk of adulteration or skin irritation in the affected area of the body. Klocke et al.,^16^ investigated the biodegradable magnesium, which influences the production processes on the biocompatibility of the machined part, and analyzed macro and micro surface properties using SEM and EDX analysis. These results are then compared to biocompatibility testing concerning cell viability and toxicity. Fu et al.,^17^ analyzed that the elemental contamination of the white layer from laser cutting was more uniformly distributed on the surface than the EDM one. Intensive heat flux in laser cutting and EDM induces thermal damage such as white layer (recast layer) and heat-affected zone (HAZ), and its properties were directly related to part performance, such as corrosion, fatigue, and wear.

The main focus of the present study is the use of a biocompatible material, specifically magnesium alloy, in LAMEDM to create multi micro electrodes. To address issues such as poor surface roughness after laser machining, HAZs, and over-cutting on the workpiece material, further research is required on biocompatible electrodes and laser micro-drilled holes. The use of magnesium alloy electrodes can help alleviate problems related to micro electrode breakage during insertion and removal, which can result in toxicity and skin irritation. The novelty of this work lies in the hybridization of laser drilling with reverse EDM, discovering a new extrusion-like formation mechanism. This approach enables the fabrication of high aspect ratio microstructures, and the capability for performing simultaneous multi-electrode fabrication, all of which provide significant advantages over conventional methods, where a single electrode is fabricated at a time.^18^

## Design

The present study is carried out on a hybrid-μEDM machine DT-110i (Mikrotools, Singapore), capable of performing sequential hybrid machining. This machine has an accuracy and resolution of ±1 μm/100 mm and 0.1 μm with RC type setup EDM, and 150 W power, with a 1070 nm wavelength fiber laser being used. The Hybrid-micro-EDM machine performs multiple conventional and non-conventional machining processes in one setup only.

**Figure 4:**
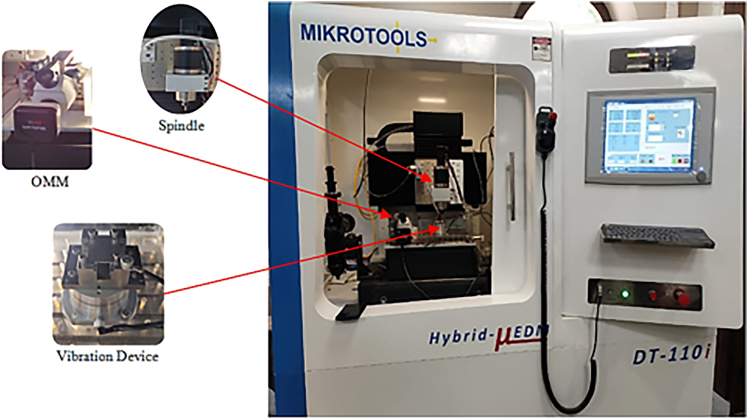
Hybrid micro-EDM machine Hybrid micro-EDM machine is used for sequential laser and micro-EDM machining. The setup includes the laser machining system, spindle, on-machine measurement unit, EDM tank, vibration device, magnetic fixture, and dielectric fluid arrangement.

The experimental setup mainly comprises a low-speed spindle (100–3000 RPM), an on-machine measurement device having an attached camera, an EDM tank, a vibration device, a magnetic fixture, and a dielectric fluid, as shown in Figure 4.

This study primarily comprises two techniques, namely, micro-EDM and Laser machining. With the help of these techniques, multiple micro-Electrodes of magnesium alloy are fabricated on a plate of Titanium material. For this experiment, the plate, as a workpiece of a thin sheet of 0.5 mm thick and 2 mm diameter magnesium alloy rod is used. The compositions of titanium and magnesium alloy were analyzed through energy dispersive spectroscopy (EDS), and some relevant property constants are given in Table 1.

**Table 1:** Elemental composition of base material titanium alloy

**Titanium alloy**				

Element	Ti	C	Zn	Al
%	96.23	3.05	0.51	0.20

**Magnesium alloy**				

Element	Mg	C	O	
%	78.86	16.31	4.82	

For this experimentation, the L9 Taguchi methodology is used with three levels of process parameters of voltage (80 V, 100 V, 120 V), capacitance (10 nf, 100 nf, and 400 nf), and vibrations (80 Hz, 100 Hz, 120 Hz). The responses were measured in terms of diameter and electrode length.

### Hybrid fabrication of micro multi-electrodes

In hybrid machining, the integration of two or more machining techniques to achieve optimal results is exemplified in this study by the utilization of sequential laser-assisted μ-EDM.^19^ The fabrication of micro electrodes serves a broad range of applications in the medical and industrial sectors. Although the electrochemical turning, similar to conventional turning and milling processes, can only produce one electrode at a time with poor dimension control and limitation of high aspect ratio,^20^ this laser-assisted hybrid micro EDM method allows for the creation of multiple micro electrodes simultaneously. The fabrication of a micro multi-electrode involves a two-stage process, starting with laser drilling and followed by the fabrication of the multi-electrode using reverse micro-EDM, as shown in schematic Figure 6. The necessary machining conditions for both laser drilling and micro-EDM are as follows.

#### Experimental conditions for laser machining

A high-intensity, high-energy beam of 150 W and a wavelength of 1070 nm from a class 4 fiber laser was used for micro-fabrication. Highly purified pressurized (UHP) nitrogen gas is used as an inert environment for assisting machining. Laser machining is a sophisticated and rapid machining method that can cut any type of material in a fraction of a second, whether it is conductive or non-conductive, as compared to micro-EDM. It is necessary to set the minimum distance for laser fire, called the stand-off distance, where the actual high-intense energy beam strikes the surface of the workpiece. In this experiment, a 0.4 mm stand-off distance was set for better machining conditions. The following parameters are used for the fabrication of micro holes: frequency (Hz), pulse width (in ms), and percentage current (% I). The current percentage (Current set point) represents a comparative percentage of the maximum acceptable power output of the laser source, as shown in Table 2.

**Table 2:** Process parameters and its ranges for laser machining

Workpiece	Process parameters	Values
Titanium Alloy	Current set point (%)	60
	Feed Rate (mm/min)	50
	Frequency (Hz)	100
	Gas Pressure (Bar)	9

**Figure 5:**
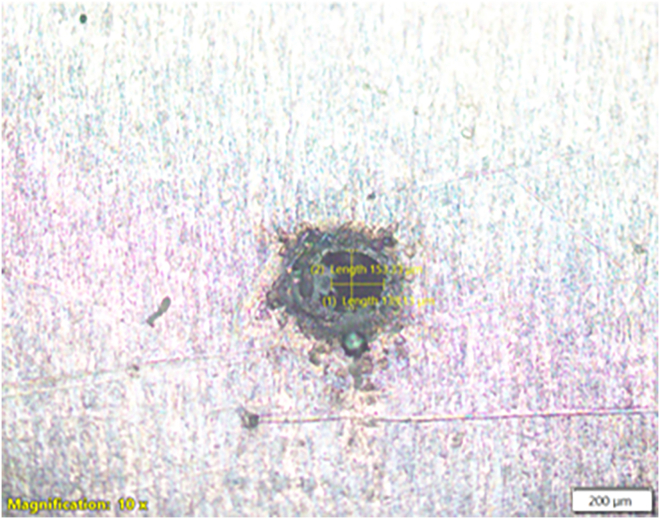
Laser-drilled micro hole Optical micrograph of a laser-drilled micro-hole fabricated on the workpiece surface. The image shows the initial micro-hole produced during the first stage of the hybrid process before reverse micro-EDM finishing. The scale bars are shown in the image.

In this particular study, a micro multi-electrode is produced through a two-stage process using LAMEDM. First, a 150-micron diameter micro hole is drilled using laser machining, as shown in Figure 5 with specific constant parameters that were selected through a pilot study, such as a current set point of 60%, Frequency of 100 Hz, gas pressure of 9 Bar, and feed rate of 50 mm/min.

#### Experimental conditions for reverse micro-EDM machining

**Figure 6:**
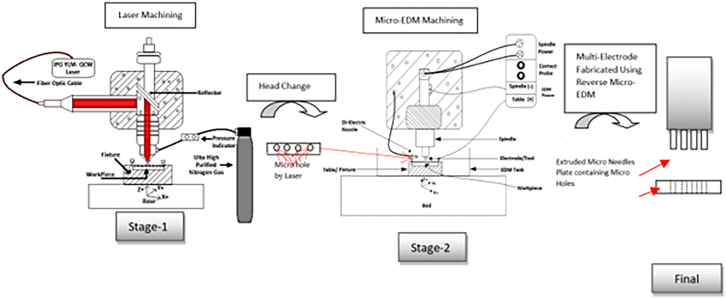
Schematic diagram of multi-electrode fabrication using the laser-assisted micro-EDM method Schematic representation of the two-stage laser-assisted micro-EDM process used for multi-electrode fabrication. In stage 1, laser machining is used to generate micro-holes. In stage 2, the machining head is changed to the micro-EDM setup, and reverse micro-EDM is used to form the multi-micro-electrode features.

**Figure 7:**
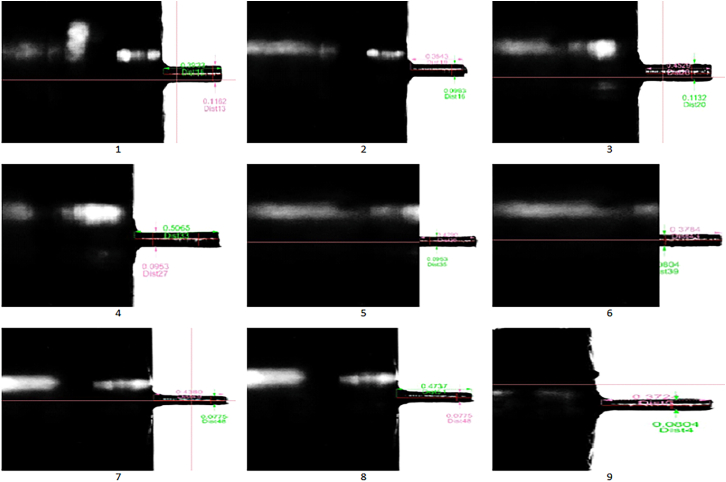
Microscopic images of rods Microscopic images show the fabricated rod-type micro-electrode features obtained under different experimental conditions. The images correspond to the Taguchi L9 experimental runs and show changes in micro-electrode diameter and length under different voltage, capacitance, and vibration conditions.

Next, the laser head is replaced with an EDM setup (R-C type), where the laser-drilled micro-hole plate functions as a tool and it becomes negatively charged, while the fixed rod in the spindle, which produces multiple electrodes, acts as the workpiece and becomes positively charged. The dielectric fluid applied is EDM Oil with a viscosity of 2.34 at (cSt) 40 °C and flash point, COC 104 °C. Dielectric is an important element for proper flushing of debris during the micro-EDM process. Furthermore, low-frequency vibration can help with the proper flushing out of debris from the machining region.^21^ In this study, process parameters utilized, such as capacitance, voltage, and the role of the vibratory device, play an important role in place of spindle rotation as shown in Table 3. It utilizes low-frequency vibrations that accelerate the machining process and also facilitates the extrusion process, which is not achievable through rotation alone, as shown in Figure 6.^22^ Due to reverse polarity material, on the electrode side is eroded more by bombardment of negatively charged electrons on positively change electrode.^23^ The electrodes are extruded from the laser-drilled micro hole such as an extrusion process during reverse micro EDM machining process, as shown in Figure 7 captured by vision measurement system (Rapid-I). The resulting multi-electrodes can be used to fabricate multiple holes using the micro EDM technique in a single process.

**Table 3:** Process parameters and its ranges for micro-EDM

Tool material	Process parameters	Ranges
Magnesium alloy	Vibration	80–120 Hz
	Capacitance	10–400 nF
	Voltage	80–120 V
	Feed rate	0.4 mm/min

## Results

**Figure 8:**
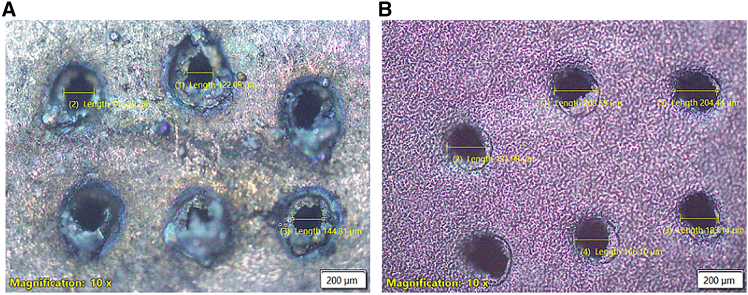
Laser micro holes and reverse EDM-treated micro holes Optical micrographs compare micro-hole morphology before and after reverse micro-EDM processing. (A) Laser-machined micro-holes show irregular geometry and thermally affected regions. (B) Micro-holes after reverse micro-EDM show improved feature definition and more controlled geometry. Scale bars are shown in the image panels.

This present study is based on selecting the workpiece as a titanium alloy, and the electrode comprises a magnesium alloy material on which the multi micro-electrodes were fabricated. The primary experiment was conducted using process parameters and values as shown in Tables 2 and 3, and the response is recorded in Table 4. During laser machining, due to thermal erosion, material gets evaporated, and machining is done in a fraction of a second, but due to this tremendous heat, with pressurized gas, a micro hole is formed and some overcuts are found, as shown in Figure 8A.

**Table 4:** Taguchi L9 array design of experiment for reverse micro EDM

Sr. No.	Voltage (V)	Capacitance (nf)	Vibration (Hz)	Diameter (mm)	Length (mm)
1.	80	10	80	0.095	0.50
2.	80	100	100	0.089	0.39
3.	80	400	120	0.080	0.41
4.	100	10	100	0.110	0.45
5.	100	100	120	0.095	0.44
6.	100	400	80	0.077	0.39
7.	120	10	120	0.110	0.49
8.	120	100	80	0.098	0.38
9.	120	400	100	0.074	0.38

**Figure 9:**
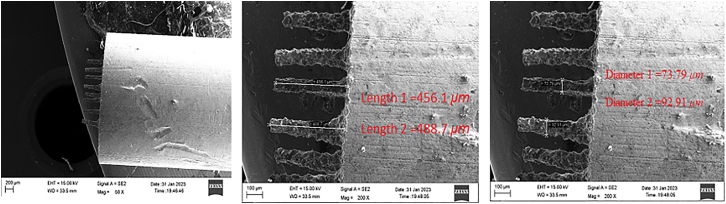
SEM images of multi-micro electrodes Scanning electron microscopy images of the fabricated multi-micro-electrodes. The measured electrode lengths were approximately 456.1 μm and 488.7 μm, while the measured diameters were approximately 73.79 μm and 92.91 μm. The images demonstrate the formation of high-aspect-ratio micro-electrode features through the hybrid process.

In electro discharge machining, a workpiece material is eroded or directly evaporated due to spark erosion in a precise region. Sometimes the electrode material gets liquefied and deposited on the inner surface of the machined region.^24^^,^^25^ In LAMEDM, which is rapid process for the fabrication micro electrodes than any traditional process. The poor surface finish in laser machining causes surface roughness of the micro electrode, as shown in Figure 9. However, the surface quality of the micro-electrodes is currently suboptimal due to the quality of the laser-drilled holes. The micro hole size of the laser drill was around 150 microns, but the extruded micro electrode size was below 100 microns by the reverse micro EDM as shown in Figures 8B and 9.

After the reverse EDM process, the electrodes get extruded into the laser-drilled micro hole, which also gets elongated as shown in Figure 7. During the reverse EDM process, as the micro electrodes travel through the laser-drilled micro hole, they are machined by spark erosion caused by the internal surface of the hole.^26^^,^^27^ It causes them to shrink such that their diameter is smaller than the original diameter of the laser-drilled micro hole, as shown in Figures 8A, 8B, and 9. This method gives the fabrication of high aspect ratio micro electrodes around 6–7 in a single go without any hindrance.

**Figure 10:**
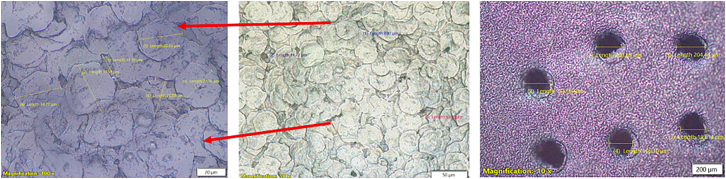
Reverse the EDM micro holes dimensions Optical micrographs show the dimensional characteristics of reverse-EDM-treated micro-hole surfaces. The images show inter-crater spacing and micro-hole geometry after reverse micro-EDM processing, indicating the surface morphology relevant to potential biomedical implant applications.

Another potential application of the reverse EDM-treated sample, as shown in Figure 10, is its use as a biomedical implant. The inter-crater spacing of approximately 30 μm promotes effective osseointegration by facilitating cell anchorage within the surface features, thereby enhancing bone-implant bonding.^28^^,^^29^

### ANOVA general linear model: Length and diameter versus voltage, vibration, capacitance

From the above ANOVA general linear model analysis for the response parameter capacitance is the most significant parameter, followed by voltage and vibrations. Also, the coefficient of determination (R-sq) is close to 1, i.e., 93.75% and 93.16% for length and diameter means model is well-fitted as shown in Tables 5 and 6.

**Table 5:** Analysis of variance for length

Source	DF	Adj SS	Adj MS	F-value	P value
Voltage	2	0.000422	0.000211	0.39	0.721
Vibration	2	0.002422	0.001211	2.22	0.310
Capacitance	2	0.013489	0.006744	12.39	0.075
Error	2	0.001089	0.000544	NA	NA

**Model summary**					

NA	S	R-sq	R-sq (adj)		NA
NA	0.0233333	93.75%	75.00%		NA

**Table 6:** Analysis of variance for diameter

Source	DF	Adj SS	Adj MS	F-value	P value
Voltage	2	0.000072	0.000036	0.75	0.571
Vibration	2	0.000042	0.000021	0.44	0.696
Capacitance	2	0.000194	0.000597	12.44	0.074
Error	2	0.000096	0.000048	NA	NA
Total	8	0.001404	NA	NA	NA

**Model summary**					

NA	S	R-sq	R-sq (adj)		NA
NA	0.0069282	93.16%	72.65%		NA

### Response optimization for length and diameter

**Figure 11:**
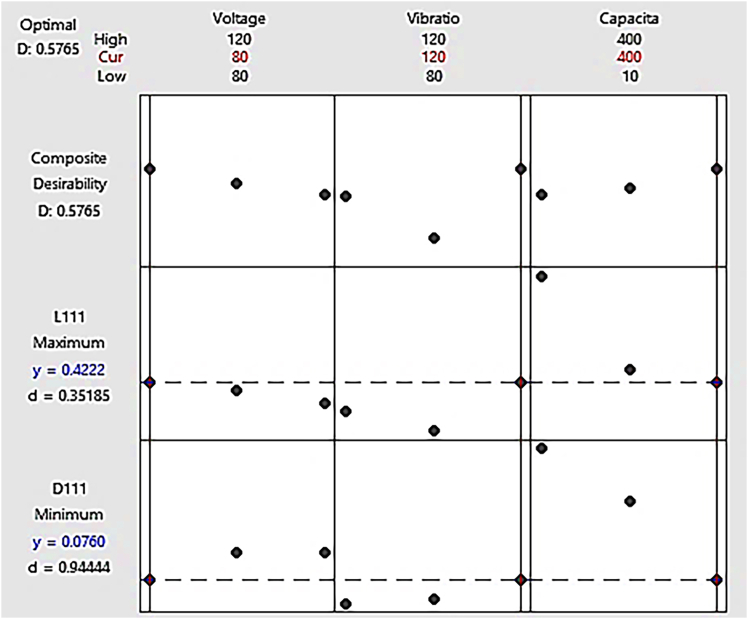
Predicted process values for length and diameter Response optimization plot shows the predicted process conditions for achieving maximum micro-electrode length and minimum diameter. The optimum predicted process parameters were 80 V voltage, 120 Hz vibration, and 400 nF capacitance, with predicted responses of 0.4222 mm length and 0.076 mm diameter.

From the response surface regression plot using Minitab software for length and diameter a multi-objective optimization obtained as shown in Figure 11. The optimum predicted process parameters for maximum Length and minimum diameter is capacitance of 400, voltage of 80 V, and vibration of 120 Hz. And on the basis of these process parameters, predicted responses are 0.4522 mm for Length and 0.076 mm for Diameter, as shown in Table 7.

**Table 7:** Predicted response optimization values

Response	Goal	Lower	Target	Upper	Weight	Importance
Length	maximum	0.38	0.500	1	1	1
Diameter	minimum	1	0.074	0.11	1	1

**Predicted parameter to get optimize response**						

**Solution**	**Voltage**	**Vibration**	**capacitance**	**length fit**	**diameter fit**	**composite desirability**
1	80	120	400	0.4222	0.076	0.576459

### Experimental validation

The predicted value came from response optimization of length and diameter is validated by drilled multiple micro holes on the titanium alloy, as shown in Figure 7. The experiment is conducted with process parameters of voltage of 80 V, capacitance of 400 nF, and vibration of 120 Hz, and the responses are measured again, i.e., Length and Diameter. The experimentally measured and predicted length and diameter values are compared by calculating mean square error (MSE) for length, i.e., 0.001156, and for diameter i.e., 0.000009, which is negligible as shown in Table 8.

**Table 8:** Validation experiment

Predicted		Experimental	
Length	diameter	length	diameter
0.422	0.076	0.456	0.073
MSE for length		MSE for diameter	
0.001156		0.000009	

### Biocompatibility assessment (cytotoxicity test)

Biocompatibility of the fabricated micro-hole structures was evaluated through an *in vitro* cytotoxicity test in accordance with ISO 10993-5 guidelines at Venture Center Pune. The assessment was performed using the direct contact method on L929 mouse fibroblast cells (NCTC clone 929, strain L), which are widely accepted for cytotoxic screening of biomaterials.^30^ This fibroblast cell helps to generate connective tissue, which is a fibrous cellular structure that aids and joins other tissues or organs in the body.^31^^,^^32^^,^^33^ Two types of samples were investigated: laser-treated micro-hole surfaces and micro-EDM (micro-EDM) treated micro-hole surfaces on magnesium alloy AZ31. The results demonstrated a notable difference in cell viability between the two fabrication techniques. The laser-treated micro-hole samples exhibited a cell viability of 78.20%, indicating moderate cytocompatibility, whereas the Micro-EDM-treated samples showed significantly higher cell viability of 96.59%, reflecting excellent cytocompatibility. According to ISO 10993-5 standards, materials exhibiting cell viability greater than 70% are considered non-cytotoxic.^34^

Therefore, both fabrication methods fall within the acceptable biocompatibility range; however, the micro-EDM processed surfaces exhibit superior biological performance. The enhanced cytocompatibility of micro-EDM treated surfaces may be attributed to reduced thermal damage, minimal surface oxidation, and improved surface integrity compared to laser processing, which can induce localized HAZs and microstructural alterations. These findings suggest that Micro-EDM is a more favorable fabrication technique for biomedical applications involving magnesium alloy AZ31, particularly where high cell viability and tissue compatibility are critical. Also, X-ray diffraction (XRD) analysis is conducted to ensure that any toxicity occurs after laser and AZ31 magnesium alloy treated micro hole.

### X-ray diffraction (XRD) analysis

**Figure 12:**
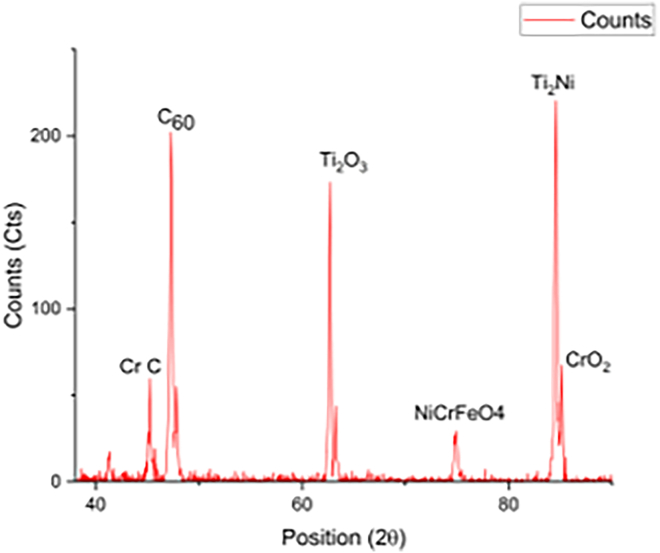
XRD graph of laser-treated micro hole X-ray diffraction pattern of the laser-treated micro-hole surface. The pattern shows phase changes and oxide formation associated with the high thermal input during laser machining, including the formation of oxide phases on the treated surface.

**Figure 13:**
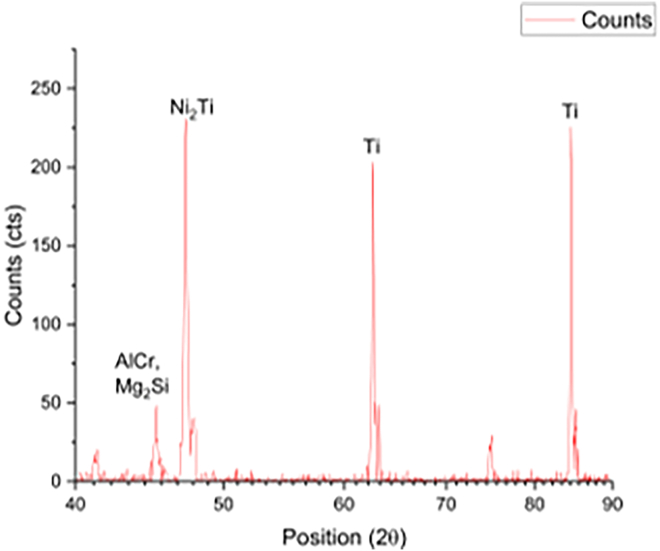
XRD graph of EDM-treated micro hole X-ray diffraction pattern of the micro-EDM-treated micro-hole surface. The pattern indicates the presence of AlCr and Mg_2_Si compounds, and no toxic phase was identified through XRD analysis, supporting the biocompatibility assessment of the EDM-treated surface.

The laser-treated micro hole contained phase change due to high temperature. Some oxide also forms like Ti_2_O_3_, CrO_2_, and NicrFeO_4_ as shown in Figure 12 of the XRD graph. Due to the immense heat generated by laser machining, a zone is produced, and a phase transition occurs. Whereas Magnesium alloy AZ31 EDM treated micro hole contained AlCr,Mg_2_Si compound, which is non-toxic and no other toxic material found through this XRD method as shown in Figure 13.^35^

## Discussion

LAMEDM offers a rapid method for fabricating multiple micro electrodes of magnesium alloy in a single operation. Magnesium alloy is a bioactive material, and when the electrodes are extruded onto a titanium alloy, the resulting product is biocompatible and has numerous medical applications. This proposed method introduces a scalable and efficient route for the batch production of micro electrode, overcoming limitations such as low throughput and complexity in existing techniques.

1. This technique allows for the manufacture of high aspect ratios up to 7 without any obstacles or breakage.
2. Moreover, the parametric study reveals that the dimensional control of micro electrodes depends mostly on capacitance of 400 nf, rather than voltage and vibrations.
3. Furthermore, *in vitro* cytotoxicity study confirmed that the cell viability of 96.59% of the micro electrode without any toxicity for biomedical applications.
4. Also, the study predicted the multi-electrode length and diameter with negligible error of 0.001156 and 0.000009.

Therefore, this work lies in the hybridization of laser drilling and reverse micro EDM, the new extrusion-like formation mechanism, the ability to achieve high aspect ratio micro structure, and the capability of simultaneous multi-micro electrode fabrication.

### Future scope

The incorporation of a parametric study could improve the surface quality of the extruded micro-electrodes. Furthermore, future research may explore the impact of electrode rotation on the surface roughness of the micro-electrodes.

## Resource availability

### Lead contact

• Requests for further information and resources should be directed to and will be fulfilled by the lead contact, Kedarnath Chaudhary (kuc21.mech@coeptech.ac.in).

### Materials availability

• This study did not generate new unique reagents.

### Data and code availability

• All data reported in this paper will be shared by the lead contact upon request.

#### Code

• This paper does not report original code.

#### Additional information

• Any additional information required to reanalyze the data reported in this paper is available from the lead contact upon request.

## Acknowledgments

The authors would like to acknowledge COEP Technological University, Pune, India for permitting them to carry out experimentation and continue their research work.

## Author contributions

V.K.: writing – review and editing, writing – original draft, investigation, acquisition, and formal analysis. B.R.: visualization, supervision, and investigation. K.C.: formal analysis and data curation.

## Declaration of interests

The authors declare no competing interests.

## STAR★Methods

### Key resources table

**Table:** 

REAGENT or RESOURCE	SOURCE	IDENTIFIER
**Experimental models: Cell lines**		

L929 Mouse fibroblast cell line	Venture Center Pune	https://www.sciencedirect.com/science/article/pii/S221282711300019X

**Software and algorithms**		

Minitab 20	Minitab	https://www.minitab.com/en-us/
Origin	Origin	https://www.originlab.com/2021

### Experimental model and study participant details

In this test L929 mouse fibroblast NCTC clone 929 strain L cell line is used for the cytotoxicity test at Venture Center Pune, Maharashtra, India, for cytotoxicity test by direct contact method. L929 cells are sensitive to a wide range of toxic substances, making them suitable for evaluating the cytotoxic effects of various compounds, chemicals, or materials.

Ethical approval was not required because the study did not involve human participants, human samples, or animal experiments and used only an established L929 mouse fibroblast cell line for *in vitro* testing. Sex- and gender-based analysis was not applicable because no human participants or animal subjects were involved. The L929 cell line was authenticated by Venture Center, Pune, India, according to the quality-control procedure followed by the testing facility. Mycoplasma contamination testing was not performed.

### Method details

The laser, μ-EDM and hybrid machined samples around 1 to 2.5 mm size is cut from the base material for cytotoxicity test which then taken to Venture center laboratory at Pune, Maharashtra, India to performed *in vitro* cytotoxicity test (ISO 10993-5) by direct contact method. Furthermore, sample extraction and testing for leachable compounds in a sample can occur concurrently with direct contact testing. The detailed procedures are as follows.

1. L929 mammalian fibroblast cells were grown to subconfluency (approximately 80% confluency) in a culture plate.
2. After verifying subconfluency, previously sterilized test materials, negative and positive control samples were carefully placed on the cell layer each of the triplicate wells. 100 μL of culture medium added to each well.
3. Plates were incubated at 37 °C with 5% CO 2 and >90% humidity for 24 h.
4. After 24 h incubation, plates were examined under phase contrast microscope for assessing changes in general morphology, vacuolization, detachment, cell lysis and membrane integrity.
5. After microscopic examination, test materials, negative and positive controls were carefully removed from the plate. Culture medium was replaced with 100 μL of fresh culture medium and then 10 μL of the MTT solution was added to each well. The plate was swirled to mix the dye and incubated in dark for 3 h at 37 °C. After 3 h incubation, formazan crystals formed were dissolved in solvent and absorbance was detected at 570 nm (ref. 690 nm) on a microplate reader.

### Quantification and statistical analysis

The design of experiment is created using Minitab 20 software and conducted the experiments accordingly. After that, the output data is recorded and analyzed using statistical method ANOVA General Linear Model. In this variance is analsyed between response parameter (output) Length and Diameter versus process parameter (input) Voltage, Vibration, Capacitance, where capacitance is identified as the most significant parameter. Also, the data of compound formation is analyzed with Origin software. The microscopic structure, and elemental weights are analyzed and measured using SEM, and biochromatic microscope.

### Additional resources

No additional information.
